# Flipping the ICF: Exploring the Interplay of Theory and the Lived Experience to Reconsider Physical Activity in Community-Dwelling People With Multiple Sclerosis

**DOI:** 10.3389/fresc.2021.710618

**Published:** 2021-10-01

**Authors:** Andrea Marjorie Stennett, Lorraine H. De Souza, Meriel Norris

**Affiliations:** ^1^Wolfson Institute of Preventative Medicine, Queen Mary University of London, London, United Kingdom; ^2^Department of Health Sciences, Brunel University London, London, United Kingdom

**Keywords:** exercise, physical activity, priorities, ICF (international classification of functioning disability and health), physiotherapy, multiple sclerosis

## Abstract

People with multiple sclerosis (MS) report lower physical activity levels and are at a risk of becoming sedentary. As such, they are at an increased risk of developing secondary health conditions associated with inactivity. This is of major public health concern. Attempts to improve the physical activity levels in people with MS remain a challenge for health professionals. One key reason might be the lack of understanding about the meanings people with MS ascribe to exercise and physical activity. This paper draws on the key findings of a three-phased interconnected mixed methods sequential explanatory study to examine the meanings of exercise and physical activity from the perspectives of people with MS and health professionals. Phase 1 used a four-round Delphi questionnaire to scope and determine the consensus of priorities for exercise and physical activity and the reasons why people with MS (*N* = 101) engaged in these activities. Phase 2 used face-to-face semistructured interviews of people with MS (*N* = 16) to explore the meanings ascribed to exercise and physical activity. Phase 3 explored the perceptions of physiotherapists (*N* = 14) about exercise and physical activity using three focus groups. Using the International Classification of Functioning, Disability, and Health as a theoretical framework to underpin this study, the authors discuss the key factors, for example, emphasis on the contextual factors, that drive decision making around exercise and physical activity participation in people with MS and explore the clinical implications to health professionals.

## Introduction

Multiple sclerosis (MS) is a progressive neurological condition of the central nervous system characterized by inflammation, demyelination, and neurodegeneration. Symptoms experienced by people with MS are varied and differ between individuals. Commonly reported symptoms include, but are not limited to, reduced mobility, fatigue, difficulty with performing activities of daily living, and reduced community participation (1–3). These symptoms are often associated with barriers to engaging in exercise and physical activity within the home and community (4, 5). For purposes of this paper, physical activity is defined as “any bodily movement produced by skeletal muscles that results in energy expenditure” [(6), p. 126] and would include domestic, occupational, and sports-related activities. Exercise is defined as “a subset of physical activity that is planned, structured and repetitive” [(6), p. 126].

In the absence of a cure and with the limitations of disease-modifying therapies to stem disability accrual (7), exercise and physical activity remain a key strategy to manage the symptoms and consequences of MS. There is strong evidence that consistently demonstrates the safety (8, 9) and beneficial effects of exercise and physical activity (8). Examples include increased strength (8, 10), balance (8, 10), mood (8), mobility (8, 11), quality of life (8, 10, 12, 13), and fatigue (14). These effects have been shown to help people with MS manage MS symptoms and cope over time with the condition (15). However, despite the well-rehearsed safety and beneficial effects of exercise and physical activity, people with MS report lower levels of physical activity (16) and are reported as being sedentary (17–19).

Many different approaches have been developed to encourage more physical activity such as, “Blue prescription” (a physiotherapy approach designed to enhance adherence with physical activity in MS) (20–22), behavioral approaches (23–25), and self-management strategies (26). Although these have shown some promise in clinical trials, they have had limited impact on sustaining physical activity levels in people with MS. This highlights a potential mismatch between the evidence base and the reality of implementation for people with MS and the health (and other) professionals who work with them. As such, there is a need to reconsider the development of programmes and strategies to not only increase but also sustain physical activity levels in people with MS. That is, creating space for a multimodal approach that on one hand understands and addresses the drivers of physical activity from the lived experience while taking into account the framework and theoretical lens through which health professionals work.

Therefore, the aim of this paper is to demonstrate the interplay of a theoretical model widely used in clinical practice to gain further insight into exercise and physical activity. The authors draw on a series of studies (15, 27, 28) carried out sequentially using a mixed methods approach to provide additional insight into the lived experiences of people with MS and using the International Classification of Functioning, Disability, and Health (ICF) (29) as a conduit to discuss the key factors that drive decision making around exercise and physical activity in community-dwelling people with MS and its implications for health professionals. Following a brief overview of the ICF, three studies unpacking the meaning of exercise and physical activity will be summarized, followed by a discussion of the key findings framed within the ICF.

## The Clinical Utility of the ICF

**Figure 1:**
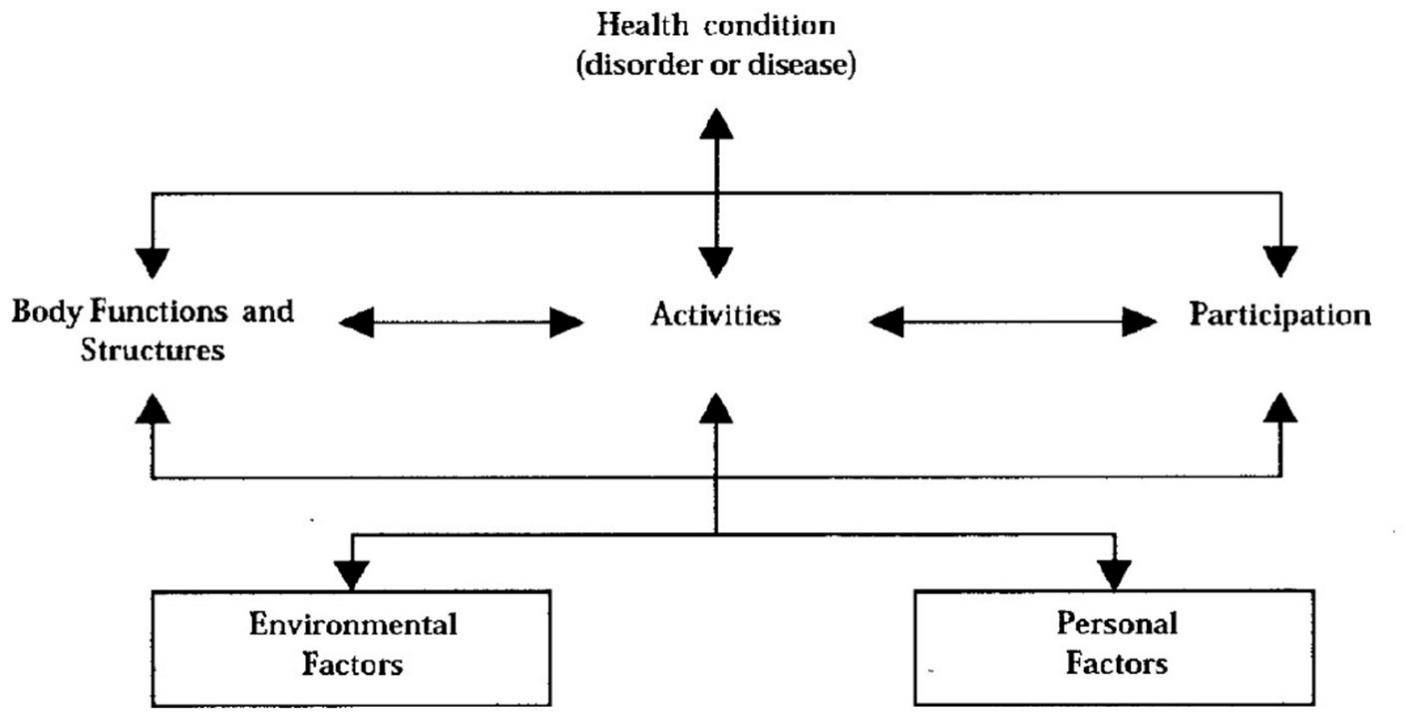
Representation of the International Classification of Functioning, Disability, and Health (29).

The ICF is a global measure that is used to understand the health and health-related status of an individual (see Figure 1) (29). It consists of two key areas, namely, functioning and disability, and contextual factors. Functioning and disability include three domains, namely, body functions and structures, activity, and participation. The contextual factors include environmental and personal factors. This model recognizes the dynamic interactions that exist between the different domains of the ICF; for example, the influence the contextual factors (environmental and personal) might have on the outcomes of an intervention (5, 30, 31).

The ICF is underpinned by the principles of the biopsychosocial approach to understanding disability. Within this context, the ICF views disability as an interaction between the biological and social aspects of life (29). It is widely used to provide a common language amongst clinicians, researchers, and people with disability, including people with MS, to describe disability and contextual factors that might have an impact on their lives (30, 32–35). Therefore, given the ethos of the ICF, which focuses on bridging the clinical and experiential gap, it is used in this study as a useful tool through which physical activity can be examined.

## The Meaning of Exercise and Physical Activity: Perspective Matters

Unpacking the meaning of exercise and physical activity is a complex issue that lends itself to an examination from different perspectives.

A three-phase mixed methods sequential explanatory design was used to determine the meanings people with MS ascribe to exercise and physical activity and its clinical implications (15, 27, 28). These studies have been published elsewhere but will be summarized here.

## Phase 1: A Day in the Life of People With MS: the Delphi Method

A four-round Delphi questionnaire scoped and determined consensus of priorities for the top 10 exercise and physical activities and the reasons people with MS were engaged in these activities (27).

A purposive sample was recruited *via* a series of targeted strategies aimed at people who had the ability to complete questionnaires, who were diagnosed with MS, and who were living in the community (*N* = 101). Data were analyzed using content analysis, descriptive statistics, and non-parametric tests.

Findings from this study provided a snapshot view of exercise and physical activity. Table 1 shows the top 10 prioritized exercise and physical activity practices and the reasons people with MS (*N* = 70) engaged in these activities. The consensus was achieved for the exercise and physical activities using Kendall's coefficient of concordance (36) (*W* = 0.744, *p* < 0.0001) and for the reasons they engaged in exercise and physical activity (*W* = 0.723, *p* < 0.0001). Overall, the exercise and physical activity practices and the reasons people with MS engaged in exercise and physical activity were diverse and highlighted the physical, psychological, and social benefits. Results indicated that unstructured activities that focused on maintaining everyday function were prioritized and had a significant impact on the identity of people with MS.

**Table 1:** Prioritised exercise and physical activity and reasons why people with MS engage in exercise and physical activity.

**Rank**	**Exercise and physical activity priorities**	**Reasons why people with MS engage in exercise and physical activity**
1	Self-care activities (e.g., shaving, shower, washing and dressing, cleaning teeth)	Improve MS symptoms (e.g., to improve or maintain strength, reduce pain, reduce spasms)
2	Every day activities (e.g., transferring, standing, pushing wheelchair or walking, climbing stairs)	Necessity (e.g., part of daily activities, activities that must be done)
3	Domestic activities (e.g., cooking, shopping, housework, laundry)	To keep active (e.g., to keep mind and body active, to maintain function and keep transferring)
4	Transportation (e.g., using public (bus/taxi) or personal transportation)	Mobility (e.g., to keep walking)
5	Leisure activities (e.g., gardening, dancing, sport, visiting friends)	Living with MS (e.g., fear of deterioration, combat MS)
6	Family Roles (e.g., spending time with family, looking after or playing with children/grandchildren)	Self-reliance (e.g., to maintain ones' independence and choice)
7	Stretches (e.g., activities done to stretch the upper and lower limbs)	Emotional wellbeing (e.g., pleasure, improve mood, reduce stress)
8	Physiotherapy (e.g., activities done with or by a physiotherapist)	Self-esteem (e.g., to manage weight, sense of achievement, self-worth)
9	Activities without weights (e.g., upper and lower limb exercises, bed and chair exercises)	Flexibility (e.g., to maintain or improve flexibility)
10	Technology (e.g., computer, exercise DVD, phone, Wii)	Social reasons (e.g., social, feel connected, be a part of community)

## Phase 2: The Meaning of Exercise and Physical Activity in People With MS: Interview Method

The meaning of exercise and physical activity was explored using in-depth semistructured, face-to-face interviews (*N* = 16; 12 women and four men). Data were analyzed using framework analysis (37). Details of the methods have been published elsewhere (15). To summarize the findings, five major themes were developed, namely, type of movement, impact of exercise and physical activity, “it changes”, sense of loss, and coping with MS. Table 2 provides a brief description of each theme. Overall, the study highlights views from the experiential perspective suggesting that people with MS took a multidimensional view of exercise and physical activity. This view of exercise and physical activity extended beyond movement; it was about using these activities as a way to cope, preserve their identity, and live life with a progressive neurological condition. Nonetheless, it was the contextual factors, such as, sense of loss and the fluctuating nature of priorities, energy demands, and choice that were dominant influences that dictated engagement or participation in exercise and physical activity.

**Table 2:** Major themes and subthemes for the meaning of exercise and physical activity.

**Main theme**	**Sub-theme**	**Description of the theme**	**Examples of quotation**
A type of movement	• Exercise as specific and organized movement • Physical activity as part of daily routine • Relevant for life with MS	Exercise and physical activity were about movement. However, the nature and purpose of each movement was different.	• Sue:…*exercise is something that, in a funny kind of way. doesn't form part of one's kind of routine… something that you make separate.time for… amm. but so, it's so… slightly more in isolation, to all the things that you would do*. (Sue, 50 years, F, severely affected, line 91–94) • *James:…physical activity I need to move from here to there and use whatever I can do, to stand up, walk, move upstairs, that, that's all physical activity to me* (James, 53 years, M, moderately affected, line 117–120)
Impact of exercise and physical activity	• Physical impact • Psychological impact • Social impact	Participants described the positive benefits of exercise and physical activity on the physical, psychological and social aspects of life.	…* feel good factor. …I don't know what it is in your body that when you exercise it sort of seems to release all these bits and pieces and it makes you feel better* (Linda, 71 years, F, moderately affected*, line 142–158)*.
It changes	• Reflections on the past and ever changing present • Uncertain future • The influence on priorities	It changes illustrate that the meaning of exercise and physical activity was contextualized to the progressive nature of MS and personal life situation.	*.you know things change so obviously …amm. exercise will change. depending on your. circumstances, ammm… as you get older you do a different type of. I mean, I'm speaking for myself. I do a different type of exercise than I would of. I also do different things now that. I've got MS, than before I had MS. (Pam, 65 years, F, Moderately affected, line 58–66)*
Sense of loss	• Loss of activity (loss of independence) • Loss of employment • Compromise and reconciliation	Participants described multiple losses. The ability to undertake certain physical activity was associated with significant loss in different areas of life such as hobbies and employment. Compromise with certain activities and a sense of reconciliation about what was loss was seen in the excepts.	*. in the past I use to love walking and would walk for hours and this is a great loss to me.I realize I can't really do the walking I use to do. (Bev, 55 years, F, moderately affected, line 90–93)*
Coping with MS	• Normalcy • Control over physical symptoms • Exercise and physical activity frames the week • Support • This is me”- identity	Participants used exercise and physical activity as a way to cope, shape and preserve their sense of self.	…*. Classes do sort of, give a framework to my week. I would think oh its x day, so x day this time I will be going to Pilates class or there would be y day. and, if I go to the Physio sort of session. I would go to that. because I am not working now if I didn't have that structure to my day… ammm… I could see the whole thing sort of falling apart! (Bev, 55 years, F, moderately affected, line 142–149)*

## Phase 3: Perceptions of Physiotherapists About Exercise and Physical Activity: FOCUS Group Method

Three focus groups were used to explore the understandings of physiotherapists about exercise and physical activity in light of the Delphi results and their relevance to clinical practice (28). Physiotherapists (*N* = 14; 12 women/2 men) with experience working with people with MS in the community were included. The focus groups were analyzed using the principles of framework analysis (37). Four themes were developed, namely, blurred terminologies, influencing factors for the meaning of exercise and physical activity, when professional expertise meets experiential expertise, and the resolve. Table 3 shows the themes and a brief description of each theme. Overall, the findings highlight the perspective of professionals that was largely shaped by training and models of practice. Physiotherapists expressed that the use of exercise is embedded into clinical practice, but physical whilst activity considered is less routine in clinical practice.

**Table 3:** Major themes and subthemes for perceptions of physiotherapists of exercise and physical activity.

**Main theme**	**Sub-theme**	**Description of themes**	**Examples of quotation**
Blurred terminologies	• Attributes of exercise and physical activity • “I kind of don't agree with my own definition”	Participants described the sense that exercise and physical activity were intricately linked. Discussions revealed attitudes toward exercise and physical activity and conflicts with the definitions used.	*.Things like walking the dog, walking to the shops, carrying the shopping. As its maybe a less intensive form of exercise (FG1, 270–274)*
Influencing factors for the meaning of exercise and physical activity	• Training vs. pragmatism in the community • External factors: Use of language, government initiatives coupled with lack of resources.	Participants discussed a number of factors that influenced the meaning of exercise and physical activity. These were described based on their training and other external factors.	*.right so we work in the NHS – you can't keep people … we're not allowed, and we can't see people every week for exercise or stretches. And mainly from a resource point of view initially, but also in terms of sort of the self- management, you know the expert patient, you know facilitating patients to manage their conditions … I think you then end up looking at exercise in a very different sort of way, cos it's not something that they're coming to you for – you are trying to encourage them to take on board the principles and then do it in their everyday life. (FG1, 462–475)*
When professional expertise meets experiential expertise	• Creation of inner tensions • Making sense of Delphi Results.	This theme reflects some of the attitudes within the study when the prioritized exercise and physical activities and the reasons why people with MS engaged in exercise and physical activity were viewed. The priorities of people with MS challenged physiotherapist understanding about the therapeutic approach used in the management of MS in the community.	*It seems ridiculous but I suppose. it wasn't how I was thinking, more than I'm surprised. I was kind of. because of the exercise thing that I conceded in my head, it was more like you know what's the most popular way to exercise rather than. more just activity. (FG2, L, 813–818)*
The resolve	• Positive reinforcement of current practice • Re-evaluation of current practice.	Through discussions, negotiations and deliberations within the group Physiotherapist attitude shifted during the focus group as they reflected on their own practice.	*See I think that one*; activities due to family role*s, I don't really address, and I think that's probably ‘cos I don't have children and my family don't live nearby. So I think that's probably something that is good to have brought up. (FG2, 921–936)*

## The Interplay of Theory and Physical Activity Using the ICF

**Figure 2:**
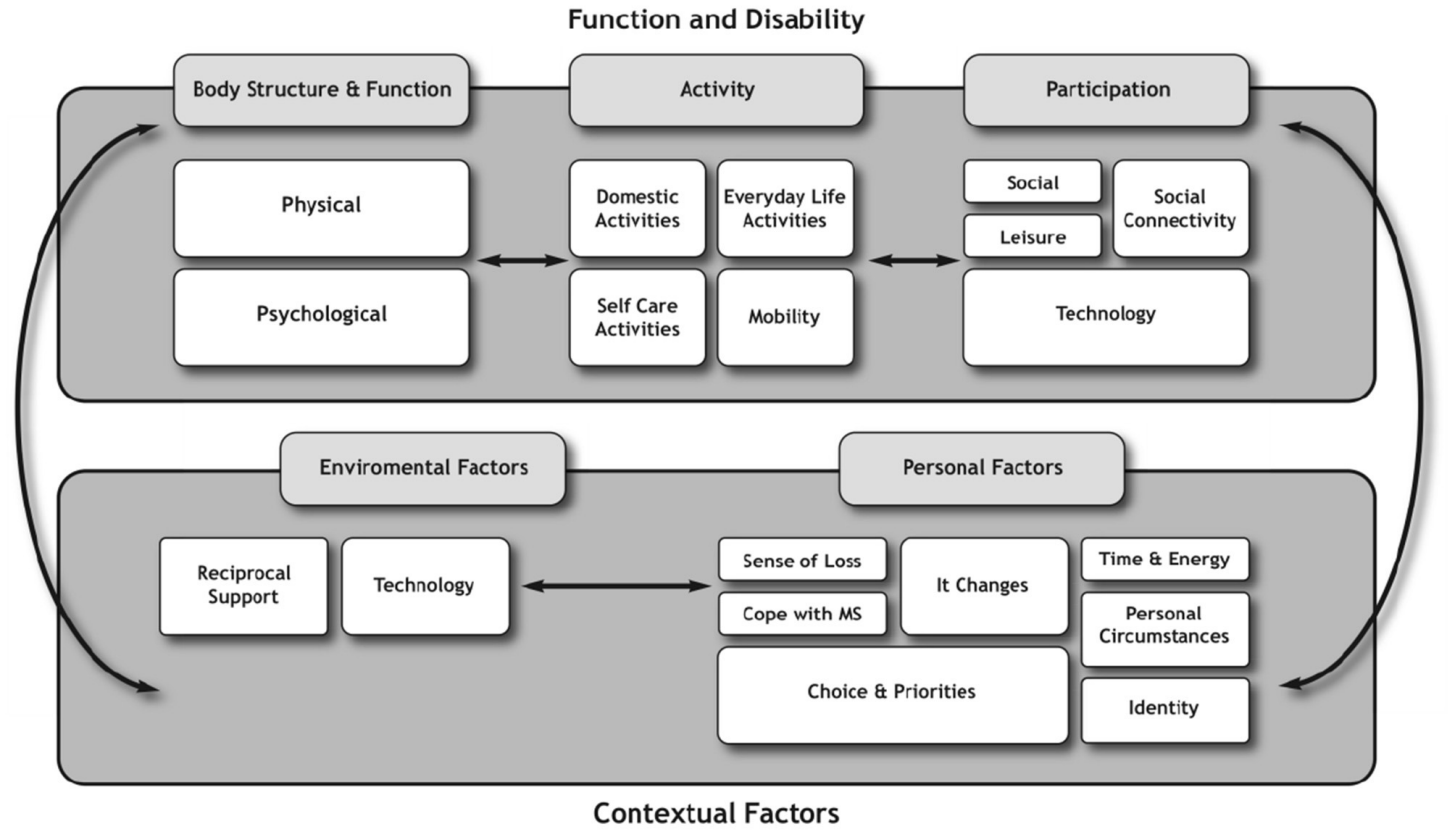
Representation of the experiential (people with MS) perspective in relation to the ICF.

**Figure 3:**
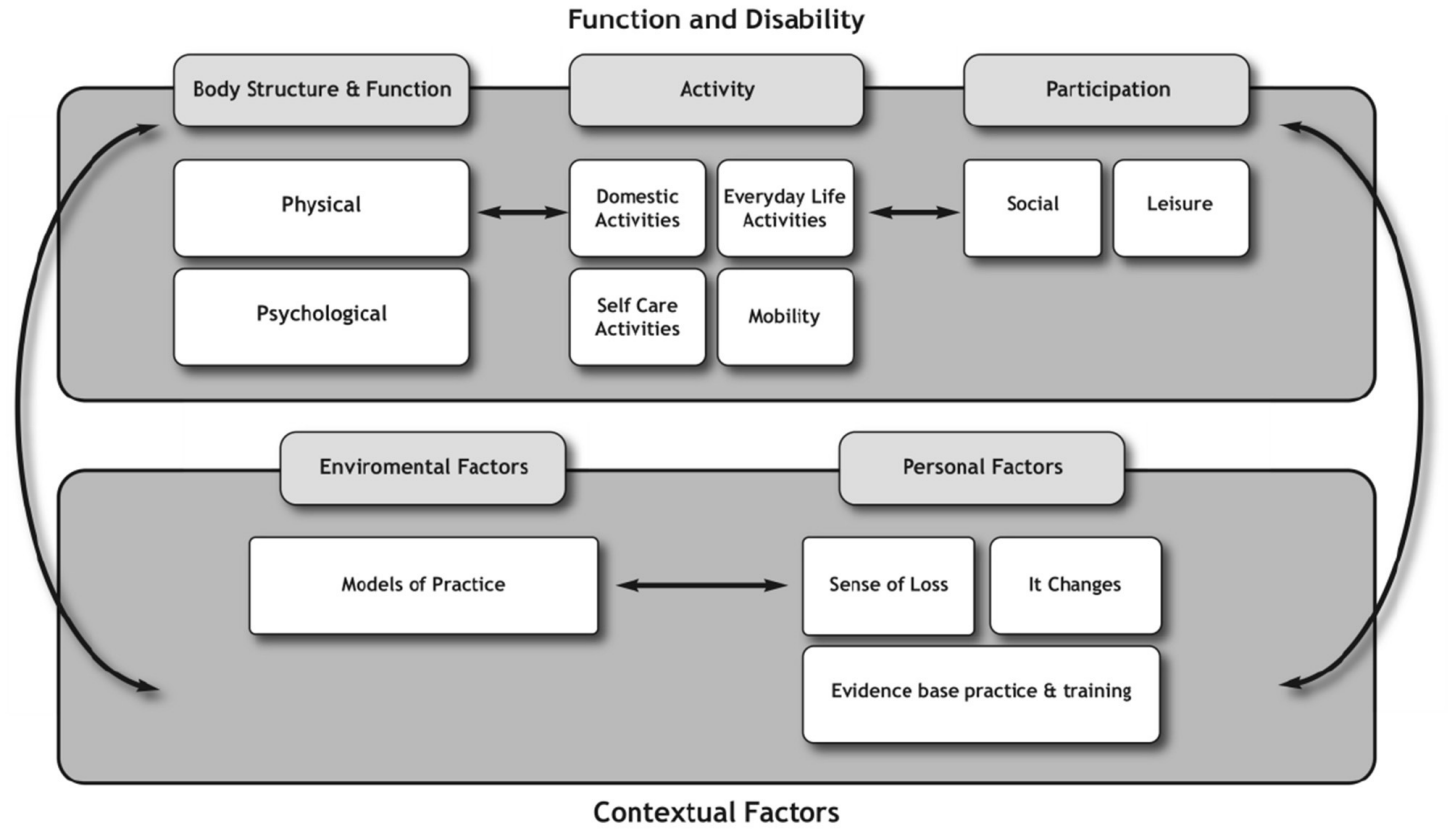
Representation of the professional (physiotherapists) perspective in relation to the ICF.

To explore the interplay of physical activity from the lived experience and the experience of professionals and how they interact with the theory, the key findings were mapped onto the domains of the ICF (29). The findings from the perspectives of people with MS (Phase 1 and 2) (see Figure 2) were mapped separately to those of the health professionals (see Figure 3).

Conceptually, the diagram highlights that the exercise and physical activity practices, and the meanings people with MS ascribed to exercise and physical activity fit within the ICF model multiple times across all of the domains of function, disability, and contextual factors. This supports the applicability of the ICF to the lived experience of people with MS in relation to exercise and physical activity.

The findings from the perspective of professionals were also mapped onto the ICF conceptually to ascertain how their views about exercise and physical activity fit within this model (see Figure 3). The representation of the perspective of physiotherapists highlights less focus on the participation and contextual factors domains. Of note, there were double the number of factors within the function and disability domain compared with the contextual domain. Indeed, the contextual factors reported by physiotherapists were less than half expressed by people with MS.

**Figure 4:**
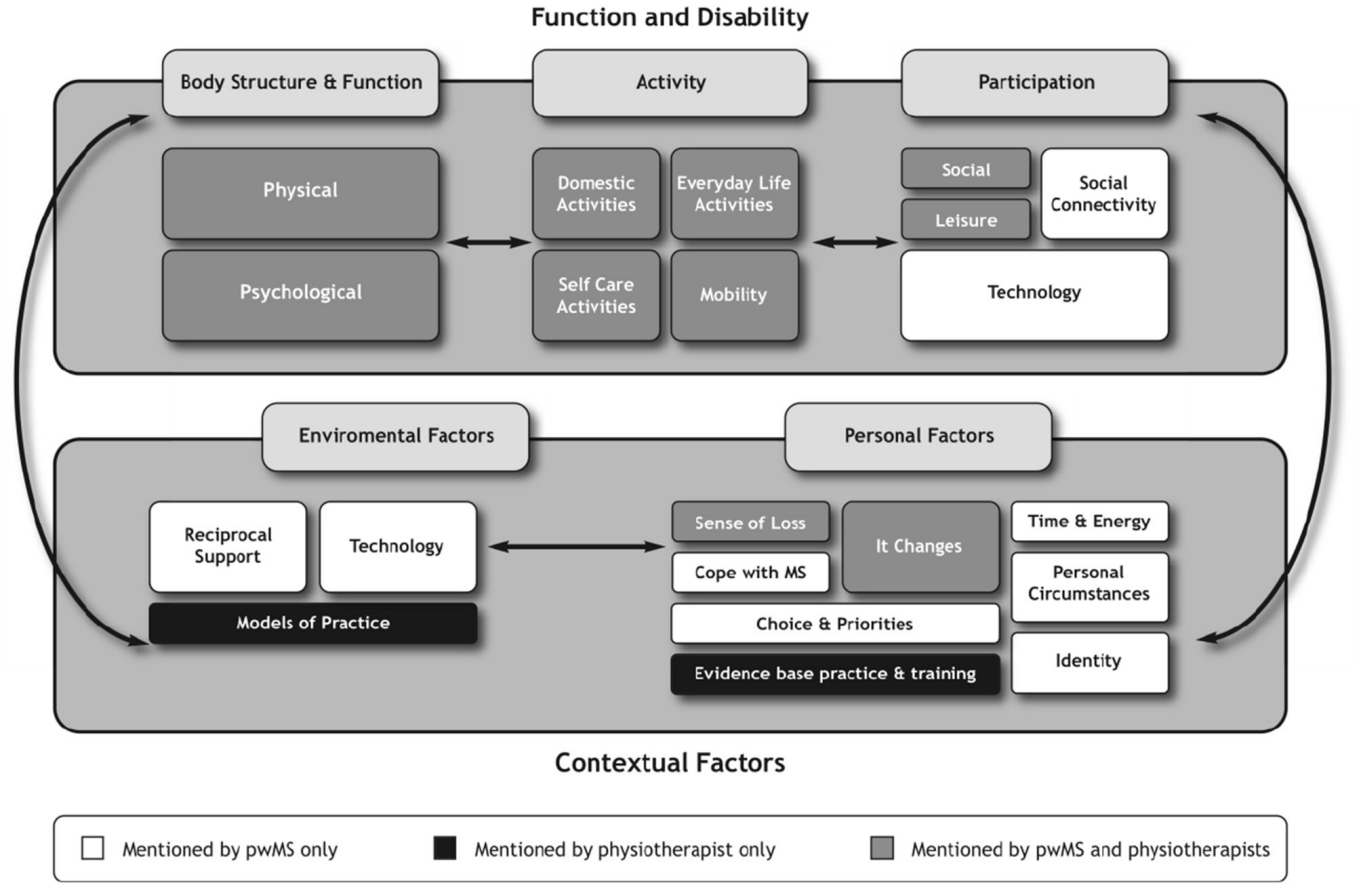
Representation of the experiential and professional perspective in relation to the ICF.

Both perspectives were then merged to compare and contrast the views of people with MS and physiotherapists (see Figure 4). It illustrates each domain and highlights that certain domains of the ICF had a greater influence on how people with MS and physiotherapists ascribed meaning to exercise and physical activity. These influences will be discussed to highlight areas of overlap and areas of dominance.

The findings from the experiential perspective demonstrated that people with MS adopted a participatory mind set, as their discussions focused on social and leisure-type activities as well as the use of technologies that enabled access to these activities. As such, they were more likely to engage in activities that connected with other people. This was in contrast to physiotherapists whose concerns revolved around whether the activities people with MS prioritized would have a direct impact on their physical performance. These findings would suggest that for physiotherapists, greater weighting and higher priority were given to the body structure, function, and activity domains rather than participation domains of the ICF in ascribing meaning to exercise and physical activity.

In contrast, the functioning and disability domains reflected a different weight of influence for people with MS compared with physiotherapists. For example, the perceptions of people with MS about exercise and physical activity were dynamic and diverse and could be reflected across all the domains of the ICF. This was exemplified by the inclusion of certain categories such as transportation and activities involving technology, which were prioritized by people with MS in the Delphi study (27). The inclusion of technology was interesting and traversed the participation and environmental domains. For example, people with MS described technology as a form of activity to facilitate higher education, as well as the use of technology as a communicative device necessary for organizing daily routines. These were not mentioned by physiotherapists, and signify the expansive views of people with MS in relation to exercise and physical activity.

Furthermore, the findings from the interviews revealed that in ascribing meaning to exercise and physical activity, people with MS were influenced predominantly by the participation and contextual factors domains; more specifically the personal factors. These contextual factors, which included the environmental and, specifically, the personal factors, shaped the perspectives of people with MS (see Figure 2), for example, “coping with MS,” “identity,” “energy demands and availability,” “time constraints,” “personal choice and priorities.” These findings concur with other researchers who have also identified that contextual factors play a significant role in influencing the other domains such as functioning and disability in people with MS as well as in people living with other forms of disability (31, 33, 35, 38). Therefore, understanding the influence contextual factors play in people with MS is important especially to health professionals who use exercise and physical activity as treatment strategies. Lack of understanding and insight into these contextual issues render people with MS seemingly inactive to the view of professionals, whereas the findings from the experiential perspective portray a different picture where people with MS are active on other priorities in other contexts.

The responses of physiotherapists did not fit neatly into the ICF framework. For example, physiotherapists shared aspects of the themes “*sense of loss” and “it changes,”* which represents the personal factors of people with MS. This finding suggests that physiotherapists do consider some aspects of the personal factors identified by people with MS. However, physiotherapists did not make the link as to how these personal factors might influence engagement in exercise and physical activity beyond the physical aspects of the life of individuals. In addition, the findings from the perspectives of physiotherapists highlight that their views about exercise and physical activity were also shaped by their own contextual factors, which were external to people with MS but influenced decisions around their management in the community. These factors included their professional knowledge based on evidence-based practice and training (personal factors) as well as models of practice, which could be represented under environmental factors.

**Figure 5:**
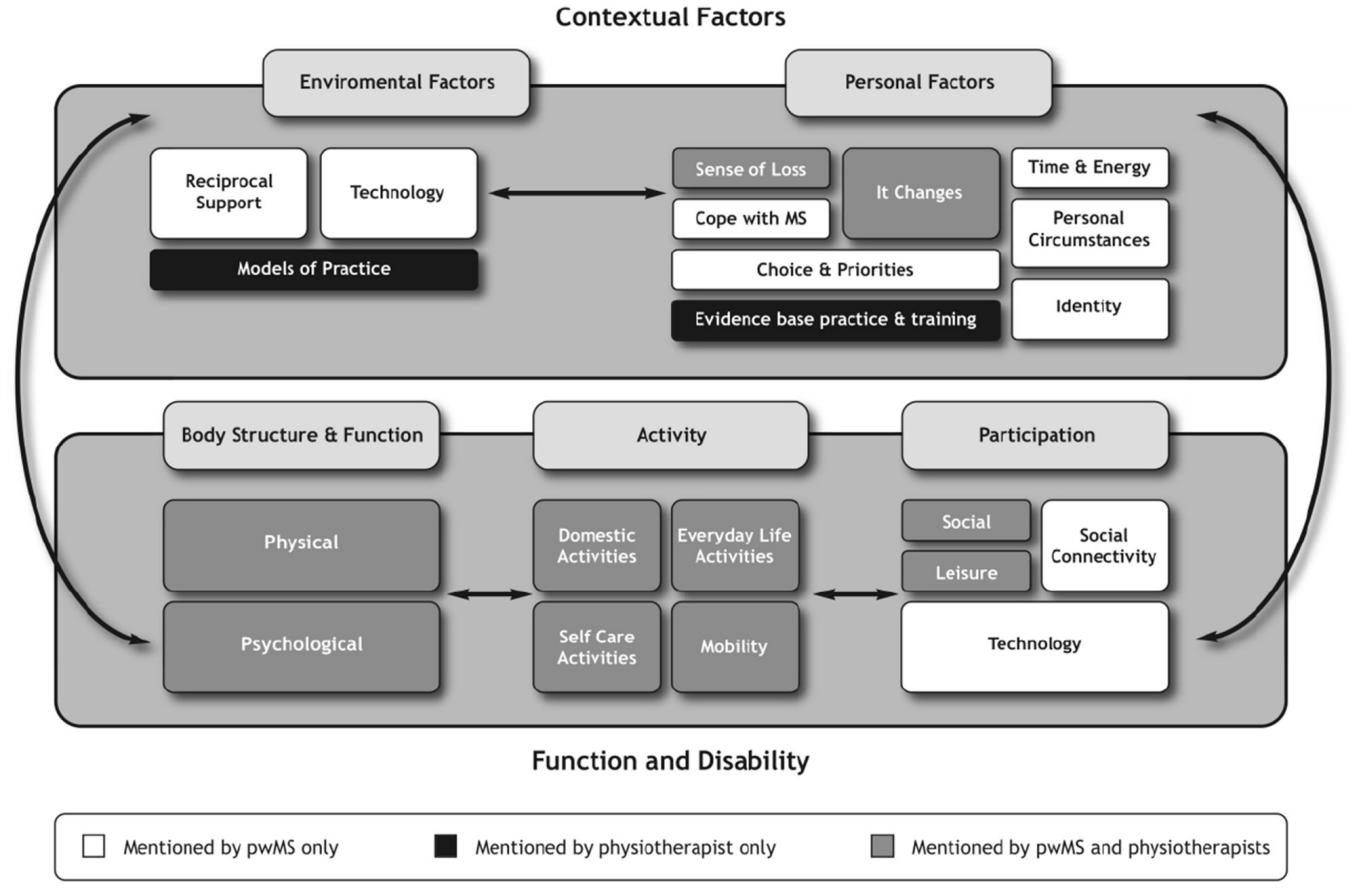
Reorientation of the ICF based on the experiential and perspective of the professionals of exercise and physical activity.

The current interpretation of the ICF implies that the contextual factors interact with the functional and disability domains (29). Although this is true, this study extended this view to also suggest that for community-dwelling people with MS, the contextual factors did not only influence the functional and disability domains but dictated what happened at the functional and disability domains. Indeed, the orientation of priority was challenged by people with MS. Having considered the views put forward by people with MS, the importance of how these views were expressed, and the heavier weight attributed by the contextual factors, the authors reconsidered the orientation of the ICF by 180°. This flip suggests that the contextual factors played a more major role than previously thought in relation to the exercise and physical activity practices, and the meanings people with MS ascribed to these practices (see Figure 5).

## Implications For Future Thinking on Physical Activity in People With MS

Reconceptualising the interactions between the contextual factors and the function and disability domains, not only as influences to be considered but also as factors having the capacity to dictate decisions about exercise and physical activity, should be central to the thinking behind engagement strategies in community-dwelling people with MS. As such, physiotherapists and other exercise professionals working in the community should give more focused attention to these domains when designing and implementing rehabilitation strategies or programs for people with MS living in the community as a way to engage and sustain exercise and physical activity in this population.

This study provided some insights about exercise and physical activity based on two sources of information, one extracted from the experiential perspective of people with MS and the other from the perspective of professionals. Exploring both sources of information suggests a “rethink” about how exercise and physical activity are viewed by health professionals and points toward taking a more person-centered approach to reflect the preferences and priorities of community-dwelling people with MS.

The key findings from the experiential perspective have not only identified the exercise and physical activity preferences and priorities of people with MS but also what these activities mean to people with MS. Having an understanding of these activities and their meanings provides some insight into the way health professionals, specifically, physiotherapists might approach exercise and physical activity in people with MS living in the community. For example, people with MS preferred engaging in exercise and physical activity practices that they valued and considered meaningful for living life with MS. This suggests that exercise and physical activity for community-dwelling people with MS were more than managing MS symptoms and also about the importance of participating in life activities, how they coped with life and maintained a sense of self. Therefore, it is now time to create more opportunities and design interventions that reflect the participatory aspect of exercise and physical activity and develop tools to monitor such interventions with a participatory focus.

## Strengths and Limitations

Using the key findings from a mixed methods study, this paper provided some insights into the intricacies associated with exercise and physical activity from the perspectives of people with MS and its clinical applicability to health care professionals. It also highlights the types of personal factors and their relevance to dictate and influence engagement in physical activity in people with MS. However, the findings must be examined, reflected on, and interpreted within the context and rigor of each study. As such, the findings might not be generalisable beyond the participants and context of the studies highlighted in this paper. Nonetheless, further research could explore the theoretical underpinnings and concepts highlighted in this study in other long-term conditions and contexts.

## Conclusions

This paper demonstrates the interplay between theory and physical activity in people with MS using the ICF model to guide discussions. The model illustrates the interaction of the ICF domains in relation to the meanings ascribed to exercise and physical activity based on the perspectives of people with MS and physiotherapists. It highlights that although people with MS were predominately influenced by participation and personal factors, physiotherapists were predominately influenced by the function and disability domains, albeit with less reference to participation. In addition, this paper adds to the existing evidence in relation to exercise and physical activity and provides evidence that the perception of exercise and physical activity in people with MS is not static and limited to any one domain within the ICF model. Instead, it highlights a complex concept, which is dynamic in nature, traversing between functioning and disability and contextual factors (personal and environmental) with personal factors having a greater influence on decisions made about exercise and physical activity in people with MS.

## Data Availability Statement

The original contributions presented in the study are included in the article/supplementary material, further inquiries can be directed to the corresponding author/s.

## Ethics Statement

The studies involving human participants were reviewed and approved by School of Health Sciences and Social Care, Brunel University. The patients/participants provided their written informed consent to participate in this study.

## Author Contributions

All authors read and contributed to the manuscript development and revision of this paper.

## Conflict of Interest

The authors declare that the research was conducted in the absence of any commercial or financial relationships that could be construed as a potential conflict of interest.

## Publisher's Note

All claims expressed in this article are solely those of the authors and do not necessarily represent those of their affiliated organizations, or those of the publisher, the editors and the reviewers. Any product that may be evaluated in this article, or claim that may be made by its manufacturer, is not guaranteed or endorsed by the publisher.
